# Comparative effectiveness of fragility fracture integrated rehabilitation management for elderly individuals after hip fracture surgery: A study protocol for a multicenter randomized controlled trial

**DOI:** 10.1097/MD.0000000000010763

**Published:** 2018-05-18

**Authors:** Sang Yoon Lee, Jaewon Beom, Bo Ryun Kim, Seung-Kyu Lim, Jae-Young Lim

**Affiliations:** a Department of Rehabilitation Medicine, Seoul National University Boramae Medical Center; b Department of Physical Medicine and Rehabilitation, Chung-Ang University Hospital, Seoul; c Department of Rehabilitation Medicine, Jeju National University Hospital, Jeju National University College of Medicine, Jeju; d Department of Rehabilitation Medicine, Gyeongsang National University Changwon Hospital, Gyeongsang National University School of Medicine, Changwon; e Department of Rehabilitation Medicine, Seoul National University Bundang Hospital, Seongnam-si, Gyeonggi-do, Republic of Korea.

**Keywords:** comprehensive health care, geriatric assessment, hip fractures, postoperative care, rehabilitation

## Abstract

**Introduction:**

Although it is essential to provide comprehensive rehabilitation after hip fracture to restore the patient to preoperative physical functioning, feasibility issues remain. Here, we describe a protocol for a randomized controlled trial (RCT) to evaluate the effectiveness of fragility fracture integrated rehabilitation management (FIRM) for elderly individuals after hip fracture surgery. We also examine the feasibility of applying FIRM in a chronic-care hospital or community-based setting.

**Methods and analysis:**

Elderly patients will be randomly assigned to either the FIRM, conventional, or control group for a 2-week intervention period following hip fracture surgery. The primary outcome of this study is Koval walking ability. All functional outcomes will be measured 1 and 3 weeks, 3, 6, and 12 months after the surgical intervention. Researchers will be blind to group allocation, and participants will be blind to outcome. A sample size of 282 participants will be necessary to demonstrate the effect of the FIRM program. After the RCT has been conducted in 3 core hospitals, FIRM will be applied in 6 community-based local hospitals to investigate the feasibility of the program. The data will be analyzed using the intention-to-treat principle.

**Trial registration number:**

NCT03430193.

## 1 Introduction

### 1.1 Background

Although the quality of the surgical and perioperative treatment of hip fracture has improved,^[1]^ the physical and functional recovery after surgery and acute care remains deficient. Before hip fracture, 11% of community-dwelling elderly individuals are bed-ridden and 16% are in long-term-care facilities.^[2]^ Within 1 year after sustaining a hip fracture, individuals experience is a very serious decrease in the quality of their life, and the mortality rate in this group is as high as 36%.^[3]^ A cohort study with the Norwegian Hip Fracture Register (n = 10,325) showed that 58% and 59% of patients had walking problems and pain, respectively, after 12 months of follow up.^[4]^ The high rates of morbidity and mortality after hip fracture have increased the cost of medical care and thus also constitute a serious socioeconomic problem. In Europe, the estimated economic burden imposed by fragility fractures in 2010 was €37 billion, with a 25% increase in the cost expected by 2025.^[5]^ Therefore, it is important to provide multidisciplinary and comprehensive rehabilitation aimed at restoring the patient to preoperative physical functioning after surgery for hip fracture.

Comprehensive rehabilitation after hip fracture usually consists of physical therapy (PT), occupational therapy (OT), fall prevention, nutritional support, psychiatric evaluation, complication care, and discharge planning with environmental modification.^[6]^ In a single-center controlled trial of hip fracture patients 70 years of age or older (n = 1077), comprehensive geriatric care was shown to improve mobility compared with the usual orthopedic care at 4 months.^[7]^ A Taiwanese study compared 3 groups of elderly patients (n = 229) with hip fracture treated with different approaches: usual care, interdisciplinary care (geriatric consultation, continuous rehabilitation, and discharge planning), and comprehensive care (interdisciplinary care plus nutrition consultation, depression management, and fall prevention).^[8]^ This research found a lower risk of depression and malnutrition in the comprehensive care group than in the interdisciplinary care group 1 year after discharge. Therefore, better functional outcomes can be expected following the provision of a comprehensive postoperative rehabilitation program to hip fracture patients.

However, there are several practical problems related to providing comprehensive rehabilitation, such as lack of facilities and human resources, which are directly linked to medical insurance coverage. Although comprehensive treatment can be readily offered at a well-established tertiary care hospital, this is not the case in community-based hospitals. Moreover, the length of hospitalization in a tertiary-care hospital after acute fracture surgery is being reduced,^[9]^ and patients who are discharged early may end up being transferred to a community-based hospital unless their functional recovery is rapid and they can be discharged home. Therefore, we should investigate the feasibility of providing comprehensive rehabilitation to hip fracture patients who are treated in a chronic or community-based setting after surgery and acute in-hospital care.

The Korean Fragility Fracture Rehabilitation Study Group has been involved in establishing a multidisciplinary hip fracture care program consisting of several orthopedic surgeons and a geriatrician. In 2016, it launched a nationwide multicenter project, funded by the Ministry of Health and Welfare, which included a fracture liaison service. The group has since developed a fragility fracture integrated rehabilitation management (FIRM) plan for patients with hip fracture, which it plans to evaluate in a pilot study^[10]^ aimed at investigating the critical rehabilitation pathway for fragility fractures in chronic-care and community-hospital settings.

### 1.2 Objectives

We aim to develop a critical pathway for FIRM and compare the effectiveness of this program with those of conventional postoperative rehabilitation and historical control treatment for individuals after hip fracture surgery. We also propose to delineate the feasibility of applying FIRM in a chronic-care or community-based hospital setting.

## 2 Methods

### 2.1 Trial design

#### 2.1.1 Stage 1: Comparison of the effectiveness of FIRM

**Figure 1:**
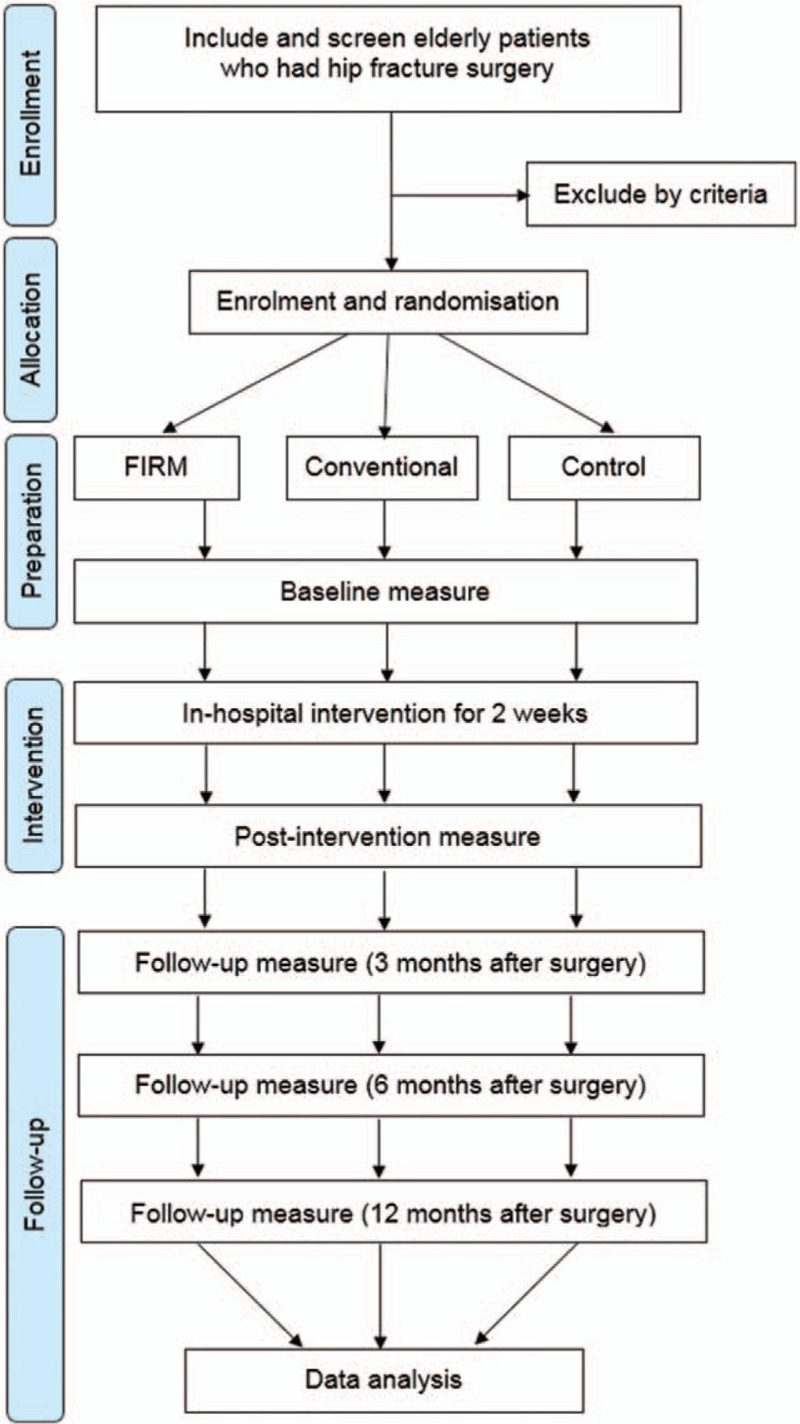
Trial procedure flow diagram.

This study was designed as a participant-blind (to outcome), researcher-blind, multicenter (Seoul National University Bundang Hospital, Chung-Ang University Hospital, and Jeju National University Hospital), randomized controlled trial (RCT) with 3 arms (FIRM, conventional, and historical control groups). During the 2-week postoperative intervention period, patients in the FIRM and conventional groups will participate in the hospital's exercise program beginning 5 to 7 days after hip fracture surgery. Only the intervention group will follow the FIRM program. Functional outcomes will be measure periodically for 12 months after surgery (Fig. 1). The trial has been registered prospectively with the Clinical Trials.gov Registry (NCT03430193) prior to participant recruitment. Important protocol modifications will be communicated to the trial registry.

#### 2.1.2 Stage 2: Feasibility of FIRM

After conducting the RCT in 3 core hospitals, the FIRM program will be tested in 6 community-based local hospitals (Chungbuk National University Hospital, Gyeongsang National University Hospital, Kyungpook National University Hospital, Presbyterian Medical Center, Dongguk University Ilsan hospital, and Chonnam National University Bitgoeul Hospital). The aim of this stage is to determine the feasibility and effectiveness of FIRM by applying the same protocol to all participating patients without randomization.

### 2.2 Participants and eligibility criteria

Elderly patients (≥65 years old) who have undergone surgery for femoral neck, intertrochanteric, or subtrochanteric fracture, regardless of surgery type (internal fixation, bipolar hemiarthroplasty, or total hip arthroplasty) will be included. Patients who have experienced the following will be excluded: hip surgery for infection, arthritis, implant loosening, or avascular necrosis; femoral shaft fracture, acetabular fracture, isolated fracture of the greater or lesser tuberosity, or periprosthetic fracture; pathologic fracture; combined multiple fracture; revision surgery; and severe cognitive dysfunction (obey command ≤ 1 step); patients who refuse to participate in a clinical trial will also be excluded. The eligibility criteria will be applied at stages 1 and 2.

### 2.3 Sample size

According to the preliminary and unpublished data of a pilot study, the mean difference and standard deviation of Koval walking ability (as a primary outcome) were 1.034 and 1.075, respectively. To detect a mean difference in the Koval walking ability grade of 0.5 for the FIRM group (0 for the conventional group and −0.5 for the control group) with at least 80% power and a significance level of 0.05 using a 2-tailed test, each group must include 85 patients (255 in total). To allow for 10% attrition, our aim is to recruit 94 patients per group (282 in total). PASS version 14.0 (NCSS, Kaysville, UT) was used to calculate the required sample size.

### 2.4 Recruitment

For the comparative clinical trial, all patients will be enrolled in 3 core hospitals. On the second day after hip fracture surgery, patients who meet the inclusion but not the exclusion criteria will be preliminarily screened by researchers in cooperation with orthopedic surgeons. Patients who agree to participate in the study will be finally enrolled. In a test of this protocol, the ratio of patient enrolments among Seoul National University Bundang Hospital, Chung-Ang University Hospital, and Jeju National University Hospital was 5:3:2 compared to the average number of hip fracture surgeries at the 3 hospitals. In stage 2, all participants will be enrolled consecutively.

### 2.5 Randomization and blinding procedure

Participants will be randomly assigned to 1 of the 3 groups using a computerized random number generator. The principal researcher will be blind to group allocation. Blinding will be initiated by a research associate implementing the randomization process described above. The associate researchers in the 3 hospitals will notify each participant's therapist of group allocation, enabling the therapist to implement the assigned rehabilitation protocol while maintaining the blinding of the other researchers. The principal researcher and the statistician will continue to be blind to group allocation, with the nominal group names assigned by the research associate maintained throughout data analysis. Outcome variables will be measured by researchers who are not involved in patient allocation. It is impossible to blind either the therapists conducting the interventions or the participants to group allocation. Nonetheless, only after their participation in the study is over will the patients be notified of the results and outcome measures.

### 2.6 Intervention

#### 2.6.1 FIRM group

FIRM is an intensive integrated care program carried out by a rehabilitation physician, physical therapist, occupational therapist, nutritionist, clinical nurse specialist, and social worker. It sets PT and OT goals, retrains physical independence, and includes detailed discharge planning (Table 1). It consists of total 10 days’ PT sessions (twice per day for 60 min) and 4 days’ OT sessions during the 2 weeks after surgery. PT (weight-bearing, strengthening, gait training, aerobic, and functional exercises) was gradually increased based on the patient's functional level. OT involving training to perform activities of daily living (ADLs; transfer, sit to stand, bed mobility, dressing, self-care retraining, and using adaptive equipment) was provided. Intensive patient education by multidisciplinary rehabilitation members was also provided.

**Table 1:**
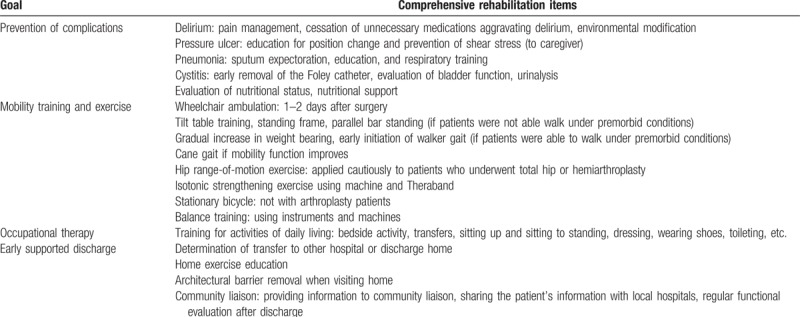
Fragility fracture integrated rehabilitation management.

#### 2.6.2 Conventional group

Conventional postoperative rehabilitation involves PT (once per day for 30 min), fall prevention, and discharge planning, including in-hospital, postoperative usual orthopedic care. The PT sessions will be the same as provided to the FIRM group.

#### 2.6.3 Control group

This group will consist of an age- and sex-matched historically controlled cohort. Participants will be discharged without having received PT during the postoperative hospitalization period. The aim will be to determine the natural course of the patients’ functional status.

### 2.7 Outcome measures

The following demographic data will be collected at baseline: age, sex, fracture location and laterality, type of surgery, and underlying disease. Functional outcomes will be measured at 1 (before intervention) and 3 weeks (after 2 weeks’ intervention), 3, 6, and 12 months after operations.

The primary outcome of this study will be Koval walking ability scores^[11]^ (range: 1–7; with a higher score indicating a worse outcome), which rates physical functioning according to walking dependency. The secondary outcomes will be as follows: physical functioning and walking ability, assessed according to the functional ambulatory category^[12]^ (range: 0–5; a lower score indicates a worse outcome) and the modified Rivermead mobility index^[13]^ (range: 0–40; a lower score indicates a worse outcome); balance and fall risk, assessed using the Berg balance scale^[14]^ (range: 0–56; a lower score indicates a worse outcome); cognition, evaluated using the Korean version of the Mini-Mental State Examination^[15]^ (range: 0–30; a lower score indicates a worse outcome); mood, evaluated using the Korean version of the Geriatric Depression Scale^[16]^ (range: 0–30; a lower score indicates a worse outcome); quality of life (QOL), evaluated using the Euro Quality of Life Questionnaire 5-dimensional classification^[17]^ (EQ-5D; range: 0–1; a lower score indicates a worse outcome); ADLs determined using the Korean version of the modified Barthel index^[18]^ (range: 0–100; a lower score indicates a worse outcome) and the Korean version of the Instrumental ADL^[19]^ (range: 0–3; a higher score indicates a worse outcome); and frailty assessed based on fatigue, resistance, ambulation, illnesses, and loss of weight (FRAIL) using the Korean version of the FRAIL scale^[20]^ (K-FRAIL; range: 0–5; a lower score indicates a worse outcome). Functional assessments will be conducted with the control group via telephone surveys performed at the same time points but measures will be limited to the Koval and EQ-5D. The adherence and drop-out rates of the program will be monitored during stage 2, in which FIRM will be applied in the community-based hospital system. Each researcher charged with outcome evaluation at each of the 3 core hospitals will work on standardizing all measuring protocols by arranging a consensus meeting before the start of the study. Regular meetings to discuss ways to unify the measurement methods will then be held.

### 2.8 Data analysis

Data will be collected using a standardized data entry form and entered into the data management system. The intention-to-treat principle will be used for data analysis. Participant characteristics will be described using means and standard deviations for continuous data and frequencies and percentages for categorical data. The 3 groups will be compared using an analysis of variance (ANOVA) or the nonparametric equivalence Kruskal–Wallis test if required. To compare paired data (intragroup) between 2 different points, we will use repeated-measures ANOVA and Friedman tests for continuous and nonparametric data, respectively. Statistical significance will be defined as a *P* value < .05. All statistical analyses will be performed using SPSS version 19.0 for Windows (IBM Corp, Chicago, IL).

### 2.9 Patient and public involvement

Patients and public were not involved in the development of the research question and the selection of outcome measures. However, this research project started with a problem-solving clinical research of the government with public interest. Because the importance of comprehensive management and rehabilitation of hip fracture patients has been recognized as a major social issue, this study can fully reflect the needs of the public.

### 2.10 Ethics and dissemination

The study will be performed in accordance with the relevant guidelines of the Declaration of Helsinki, 1964, as amended in Tokyo, 1975; Venice, 1983; Hong Kong, 1989; and Somerset West, 1996.^[21]^ Written informed consent for all interventions and examinations will be obtained at patient admission. The Ethics Board will be informed of all serious adverse events and any unanticipated adverse effects that occur during the study. The study protocol has been registered at Clinicaltrials.gov and will be updated. The study methods are in accordance with the SPIRIT guidelines for reporting randomized trials.^[22]^ Direct access to the source data will be provided for monitoring, audits, Research Ethics Committee/IRB review, and regulatory authority inspections during and after the study. All patient information will be coded anonymously, with only the study team having access to the original data. The study results will be disseminated in peer-reviewed publications and conference presentations.

## 3 Discussion

Loss of functional independence, which is a long-term sequela of hip fracture,^[23]^ is often accompanied by a deterioration in health-related QOL.^[24]^ Longitudinal studies have shown a decline in the functional status of patients with hip fractures following reductions in rehabilitation services.^[25]^ Conversely, the long-term functional outcomes of patients who received comprehensive rehabilitation in recent clinical trials performed after hip fracture surgery included significantly better mobility and ADL scores.^[7,26,27]^ However, the dissemination of a well-designed rehabilitation program to community-based hospitals is challenging. Our study examines the efficacy of FIRM, a postoperative comprehensive rehabilitation program, and investigates the feasibility of its use in community hospitals with the goal of subsequently implementing it and evaluating its efficacy on a nationwide basis to confirm the findings.

## Author contributions

Jae-Young Lim conceived the study and is the principal investigator. Sang Yoon Lee, Jaewon Beom, Bo Ryun Kim, and Seung-Kyu Lim contributed to the conception of the study. All authors approved the version to be published and are responsible for its accuracy.

**Conceptualization:** Jae-young Lim.

**Data curation:** Jaewon Beom, Bo Ryun Kim, Seung-Kyu Lim.

**Formal analysis:** Jaewon Beom, Bo Ryun Kim, Seung-Kyu Lim.

**Funding acquisition:** Jae-young Lim.

**Investigation:** Sang Yoon Lee, Jaewon Beom, Bo Ryun Kim, Jae-young Lim.

**Methodology:** Sang Yoon Lee, Bo Ryun Kim, Seung-Kyu Lim.

**Project administration:** Jae-young Lim.

**Resources:** Seung-Kyu Lim.

**Supervision:** Jae-young Lim.

**Validation:** Jae-young Lim.

**Writing – original draft:** Sang Yoon Lee, Jae-young Lim.

**Writing – review & editing:** Sang Yoon Lee, Jaewon Beom, Bo Ryun Kim, Seung-Kyu Lim, Jae-young Lim.
